# Contribution of Gamma-Aminobutyric Amino Acid and Free Amino Acids to Low-Salt Whole-Wheat Bread through the Addition of Spice Extracts—An Approach Based on Taste Quality

**DOI:** 10.3390/foods13121900

**Published:** 2024-06-17

**Authors:** Kumiko Hisaki, Chikae Sakamoto, Hina Matsui, Hiroshi Ueno, Yukiko Ueda

**Affiliations:** 1 Department of Nutrition, Osaka International College, Moriguchi 570-8555, Japan; 2 Department of Design for Contemporary Life, Kyoto Bunkyo Junior College, Uji 611-0041, Japan; c-saka@po.kbu.ac.jp; 3 Department of Food and Agricultural Science, Graduate School of Agriculture, Ryukoku University, Otsu 520-2194, Japana20022@mail.ryukoku.ac.jp (Y.U.); 4 Research Institute for Food and Agriculture, Ryukoku University, Otsu 520-2194, Japan; hueno@cc.nara-wu.ac.jp; 5 Department of Food Science and Human Nutrition, Faculty of Agriculture, Ryukoku University, Otsu 520-2194, Japan

**Keywords:** low-salt whole-wheat bread, spice extracts, γ-aminobutyric acid, free amino acids

## Abstract

Given the link between excessive salt consumption and hypertension, reducing salt levels in bread, an important staple food in Japan, is essential. γ-Aminobutyric acid (GABA) has a salty taste-enhancing effect in vivo, and its production is influenced by the type of spice extract in vitro. However, the effects of spices on GABA levels, total free amino acid composition, and taste quality in whole-wheat bread remain unclear. Therefore, this study aimed to investigate whether the addition of spice extracts, which do not affect bread flavor and taste, can increase the GABA level in low-salt whole-wheat bread and whether free amino acid content affects the taste quality of bread using an automatic home bread maker. Through free amino acid composition analysis and sensory testing, we evaluated the influence of six spice extracts on the composition of free amino acids, including GABA, in whole-wheat bread. We found that cumin and anise extracts were effective in increasing the GABA level to approximately twice that in whole-wheat bread. Moreover, both the preference and saltiness of the bread were favorable, indicating that these extracts are useful for reducing the salt content of whole-wheat bread. This study provides a theoretical basis for guiding industrial production.

## 1. Introduction

In Japan, bread is an important staple food, ranking second only to rice in terms of daily intake by cereal group [1]. Flour is classified according to its protein content as light, medium, or strong. Strong flours, with high protein content and gluten-forming capacity, are generally used for bread production. However, the production of bread requires salt to form gluten, and 100 g of commercial bread contains the equivalent of 1.2 g of salt [2]. The higher the bread consumption frequency, the higher the salt intake level.

It is widely accepted that reduced dietary salt intake can lower the risk of hypertension [3]. The Dietary Reference Intakes for Japanese (2020) set the target intake of salt equivalents at less than 7.5 and 6.5 g per day for men and women, respectively. The recommended salt equivalent intake to prevent hypertension and reduce the severity of chronic kidney disease is less than 6.0 g per day for both men and women. Therefore, reducing the salt level of bread is essential. However, reducing salt intake is challenging as salt enhances the taste of foods. Therefore, it is desirable to seek alternative approaches that improve taste quality in a low-salt diet without compromising the taste.

Spices are often used to reduce salt intake. Studies have shown the effectiveness of reducing salt intake by adding spices in low-salt tomato soup during cooking [4] and in hospital food services [5]. Similarly, spices such as green perilla and yuzu peel are used in Japanese cuisine to reduce salt intake [6]. Moreover, spices such as rosemary, celery seed (celery), anise, cumin, and mace reportedly have saltiness-enhancing effects and are thus expected to be useful in cooking and processed foods [7,8]. However, the type and quantity of spices used can greatly affect the flavor profile of dishes. Hence, maintaining the flavor of processed foods and dishes while reducing salt intake is essential.

In the present study, we focused on γ-aminobutyric acid (GABA)-mediated low-salt diets with a small amount of spice extract that does not affect flavor and taste [9]. Available evidence indicates that GABA helps reduce salt intake. GABA, which is synthesized by glutamate decarboxylase (GAD) using glutamate as a substrate, is a non-protein amino acid. GABA is abundant as a major inhibitory neurotransmitter in the central nervous system of humans and other mammals and has attracted attention as a functional ingredient because of its blood pressure-lowering effect. Notably, the Food Safety Commission of Japan has evaluated Foods for Specified Health Uses containing GABA as an active ingredient, recommending an intake of 10 mg or more per day for individuals with elevated blood pressure. The Commission also states that 10–80 mg of GAPA per day is safe if consumed appropriately [10]. GABA is produced in type III taste bud cells, which are also equipped with GABA-gated chloride ion channels [11]. Using taste sensation tests with human participants, GABA has been found to be perceived as a sour compound. Furthermore, adding a small amount of GABA to a salty solution increases saltiness with little change in taste quality [12]. Hence, GABA is expected to prevent high blood pressure and serve as a saltiness enhancer.

With increasing health consciousness, breads made from whole-wheat flour are also commercially sold and considered to be superior to those made from strong flours in terms of providing minerals, such as potassium, magnesium, iron, and zinc, as well as B vitamins and fiber [2]. GABA production can also be expected in these breads, as wheat germ contains endogenous GAD [13], which synthesizes GABA. Moreover, the activity of GAD65 and GAD67, isoforms of GAD, is reported to be affected by spice extracts [8].

The taste of bread may depend on the composition of its ingredients, including the type of flour and free amino acids produced during kneading, fermentation, and baking [14]. In particular, whole-grain products are generally associated with negative sensory characteristics, owing to bitter and astringent tastes [15]. Tryptophan has been identified as one of the bitter compounds in whole-grain breads [16]. However, it remains unclear whether the addition of spice extracts to whole-wheat bread dough improves the salty effect and taste quality of the finished product.

This study focused on reducing the salt content of nutrient-rich whole-wheat breads by (1) determining whether the addition of a small amount of spice extract to low-salt whole-wheat bread increases GABA levels in the bread, and (2) investigating the role of free amino acids, including GABA, on the taste quality of bread. Particularly, this study aimed to examine whether GABA and other free amino acids are produced by the spice extracts added to whole-wheat bread dough and determine the relationship between the taste of free amino acids and the taste quality of whole-wheat bread. To this end, we evaluated the influence of spices on the free amino acid composition of whole-wheat bread using sensory testing and free amino acid composition analysis.

## 2. Materials and Methods

### 2.1. Sample Preparation

#### 2.1.1. Spice Extracts

Among the spices suggested in the previous studies [8,9,12], we selected celery, anise, and cumin, which enhance saltiness; paprika, which has a minimal effect on saltiness; and lemongrass and oregano, which reduce saltiness. Anise, celery, paprika, oregano, lemongrass (Otsuya Shoten Co., Ltd., Otsu, Japan), and cumin (Mascot Foods Co., Ltd., Tokyo, Japan) powders were purchased and used in the experiments. To each spice powder (g), distilled water was added in a 1:5 or 1:10 ratio (approximately 5–10 times the weight of the spice powder). The mixture was allowed to stand overnight at 4–6 °C, stirred, and subsequently centrifuged (10,000× *g*, 5 min). The supernatant was then used as the spice extract. For bread preparation, the amount of spice extract added to 100 g of flour was equal to 0.02% of the spice powder.

#### 2.1.2. Low-Salt Bread Preparation

Low-salt bread was prepared according to the method used in a previous study [9]. Dry yeast (0.6%; Saf-instant^®^ RED, LESAFFRE, Marcq-en-Barœul, France), 1.0% salt (refined salt; The Salt Industry Center of Japan, Tokyo, Japan), 6.8% granulated sugar (Mitsui DM Sugar Co., Ltd., Tokyo, Japan), 2.0% skim milk (Pioneer Planning Co., Yokohama, Japan), 6.0% unsalted butter (Yotsuba Butter, Yotsuba Dairy Industry Co., Sapporo, Japan), and 76% water (with or without spice extract) for 100 g of flour (strong flour: Kitanokaori [12.5% ± 1.0% protein, 0.50% ± 0.05% ash], whole-wheat flour: Kitanokaori [14.0% ± 2.0% protein, less than 1.80% ash] [17]; both from Hokkaido, Tomizawa Shoten, Tokyo, Japan). Kitanokaori is a hard, red winter wheat variety characterized by a slightly higher ash content than that of foreign bread flours (Figure S1).

The ingredients were prepared at 2.5 times the amount of 100 g flour, and the bread was prepared using an automatic bread maker for the home (SD-BMT1000; Panasonic, Kadoma, Japan) in a 4 h process from kneading to the completion of baking (menu No. 4 on the machine) [18]. After baking, the bread was incubated at 26 °C for 1 h, placed in a zippered plastic bag (Ziploc; Asahi Kasei Home Products Corp., Tokyo, Japan), and stored overnight at 26 °C. Hereafter, the bread without the spice extract is referred to as the “control bread”, and the bread with the spice extract is referred to as the “spice-added bread”.

### 2.2. Amino Acid Analysis

#### 2.2.1. Sample Preparation

One gram each of the spice powder and the baked bread core were accurately weighed, and 3.5 mL HEPES-Na buffer (100 mM, pH 7.0) was added. Thereafter, it was crushed on ice (Tissue-Tearor; BioSpec Products, Inc., Bartlesville, OK, USA) and centrifuged (10,000× *g*, 10 min). To the supernatant, 60% perchloric acid was added, allowed to stand on ice (10 min), and then centrifuged (10,000× *g*, 10 min) [protein removal]. The supernatant was used as the sample for amino acid analysis.

#### 2.2.2. Amino Acid Standard Solution

GABA, hydroxyproline, glutamine, asparagine, tryptophan, and cysteine were added to Amino Acid Mixture Standard Solution Type H (Fujifilm [formerly Wako Pure Chemicals] Corporation, Osaka, Japan) containing 17 amino acids and prepared at 100 μmol/L.

#### 2.2.3. Amino Acid Analysis for Free Amino Acids

Derivatization was performed using a reaction reagent kit for ultra-fast amino acid analysis (Hitachi High-Tech Corporation, Tokyo, Japan: derivatization reagent NBD-F) according to the method reported by Ito et al. [19]. Amino acids were identified and quantified using an ultra-fast amino acid analyzer (HITACHI LaChrom Ultra; Hitachi High-Tech Corporation, Tokyo, Japan). The results are expressed as mg of free amino acids per 100 g of bread.

#### 2.2.4. Free Amino Acid Categorization According to Taste Quality

Free amino acids in food can be classified into several categories based on their primary taste [12,20,21,22,23,24,25]. Therefore, GABA is classified as an acidity and saltiness enhancer. Glutamate and aspartate are classified as having umami. Proline, glutamine, alanine, threonine, glycine, serine, and hydroxyproline are classified as sweet. Cysteine, arginine, lysine, histidine, valine, phenylalanine, isoleucine, methionine, tryptophan, and leucine are classified as bitter (Table 1).

### 2.3. Sensory Evaluation

The participants in the sensory evaluation were undergraduate students of the Department of Food and Nutrition, Faculty of Agriculture, Ryukoku University, who have experience in sensory testing. The participants provided verbal informed consent to participate in the study and acknowledged that they could not be identified via details provided in the manuscript. We have anonymized their details and do not identify them in any way. All mandatory laboratory health and safety procedures were adhered to during this evaluation. Approval was obtained from the ethical review committees of Osaka International College (No. 19-09) and Ryukoku University (No. 2023-14). The participants underwent pretesting for taste sensitivity, which involved assessing their responses to solutions with varying salt concentrations (0.04%, 0.08%, 0.16%, 0.31%, and 0.63%). Individuals whose cognitive threshold (the concentration at which they first perceived saltiness) was determined to be 0.63% were excluded from the study. Those determined to have a normal taste perception were included, resulting in 25 participants for the strong flour bread and 24 for the whole-wheat bread. Owing to incomplete responses, however, 23 and 24 participants were included for strong flour and whole-wheat bread analyses, respectively. The bread used for the sensory tests was cut from the browned part of the loaf into squares of approximately 2–3 cm before testing.

The tests were carried out in the cooking practice room at Ryukoku University. As conditions for participation, panelists were required to have a normal sense of taste and smell, not to eat or drink for one hour prior to the evaluation, and to refrain from using perfume or strong-smelling hairdressing products. Prior to sensory testing, the participants were instructed to rinse their mouths with water, take a sip of water, and taste food thoroughly throughout the oral cavity. Whole-wheat breads were evaluated based on four basic tastes (sweet, sour, salty, and umami) and overall palatability. The four basic tastes were evaluated at seven levels as follows: very strong (+3), strong (+2), slightly strong (+1), no change (0), slightly weak (−1), weak (−2), and very weak (−3). For the overall evaluation, a seven-point scoring system was used as follows: very tasty (+3), tasty (+2), somewhat tasty (+1), no change (0), somewhat bad (−1), bad (−2), and very bad (−3). Preference for the strong flour and whole-wheat breads was rated on a three-point scale as follows: “like”, “neither like nor dislike”, and “dislike” compared with that for the control bread.

### 2.4. Statistical Analysis

Statistical analysis was performed using EZR on R Commander version 1.63 [26,27] and Excel Microsoft 365 statistical functions. The relationship between free amino acids and taste quality was analyzed using regression analysis, and the R-squared value was calculated. Preferences with and without spice extract addition were compared using the Kruskal–Wallis test, with a significance level of *p* < 0.05.

## 3. Results

### 3.1. Weight, Salt Content, and Flavor of Bread

The strong flour bread weighed 420.3 ± 3.5 g on average and the whole-wheat bread weighed 408.6 ± 6.9 g, and the addition of spices did not affect the weight. Both types of bread had a salt content of 0.6 g per 100 g, which was 50% less than that of commercial bread. The effect of the spice flavor on the spice-added bread was negligible, although some participants did perceive a subtle spice-like flavor.

### 3.2. Changes in Free Amino Acid Content

#### 3.2.1. Total Free Amino Acid Content of Spice Extracts

The total amino acid contents (*n* = 3, means ± SE) per 100 g of celery, anise, cumin, paprika, lemongrass, and oregano powder were 300.0 ± 31.5 mg, 189.4 ± 5.6 mg, 153.7 ± 1.3 mg, 476.8 ± 24.7 mg, 201.4 ± 5.9 mg, and 130.0 ± 22.2 mg, respectively. Thus, the total amount of free amino acids equivalent to 0.02 g of spice powder per 100 g of flour was 0–0.1 mg, and the free amino acids in the spices did not affect the free amino acid composition of the bread.

#### 3.2.2. Free Amino Acid Content of the Control Bread According to Taste Quality

Table 2 lists the free amino acid content of the strong flour and whole-wheat breads. Whole-wheat bread contained 1.6 times more free amino acids than strong flour bread and was characterized by the highest ratio of tryptophan, followed by GABA. Tryptophan, which imparts a bitter taste, presented a content of 2.05 mg, which was 25.9 times higher than that of strong flour bread. This was followed by GABA at 1.47 mg, which was six times higher than that of whole-wheat bread. The glutamate content of 2.21 mg in strong flour bread was 0.7 times lower than that of whole-wheat bread. In terms of amino acids by taste quality, glutamate characterized the umami taste of whole-wheat bread, while proline and glutamine characterized the sweet taste, and cysteine and arginine characterized the bitter taste. This trend was also observed for strong flour bread.

#### 3.2.3. Free Amino Acid Content of Spice-Added Bread According to Taste Quality

**Figure 1:**
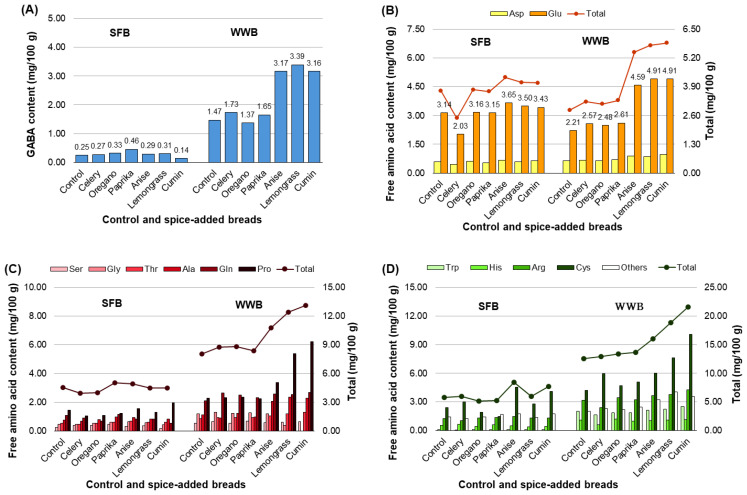
Free amino acid contents of breads. (**A**) GABA, (**B**) umami amino acid, (**C**) sweet amino acid, and (**D**) bitter amino acid contents. SFB: strong flour bread (*n* = 3), WWB: whole-wheat bread (*n* = 3). GABA, γ-aminobutyric acid; Asp, aspartate; Glu, glutamate; Ser, serine; Gly, glycine; Thr, threonine; Ala, alanine; Gln, glutamine; Pro, proline, Trp, tryptophan; His, histidine; Arg, arginine; Cys, cysteine.

Changes in the amino acid content of the strong flour and whole-wheat breads with the addition of spices are shown in Figure 1A–D. The addition of anise, lemongrass, and cumin to the whole-wheat flour increased the amount of GABA (3.16–3.39 mg) to approximately twice that of the control bread (1.47 mg; Figure 1A), reaching approximately 30% of the daily level recommended for functionality against hypertension [10]. Adding spices to strong flour did not increase the amount of GABA. Aspartate content in either strong or whole-wheat flour bread was not affected by the addition of spices, whereas glutamate content was approximately doubled by the addition of anise, lemongrass, and cumin to whole-wheat flour bread (Figure 1B), almost reaching threshold levels (Table 1). In terms of sweetness (Figure 1C), the added spices exhibited no effect on strong flour bread. In contrast, the addition of anise, lemongrass, and cumin increased the sweet amino acid content in whole-wheat flour bread. For instance, the addition of lemongrass and cumin to the whole-wheat flour increased the proline content up to (5.37 and 6.22 mg, respectively) approximately 2.5 times that of the control bread (2.27 mg); however, the proline content did not reach the threshold (Table 1). In terms of bitterness (Figure 1D), the addition of anise and cumin increased the cysteine content in both the strong and whole-wheat flour breads; however, the cumin content in both bread types did not reach the threshold (Table 1). The amount of tryptophan, which was identified as one of the factors contributing to the bitterness of whole-wheat bread [16], did not change with the addition of spices.

### 3.3. Effect of Free Amino Acids on Taste Quality

**Figure 2:**
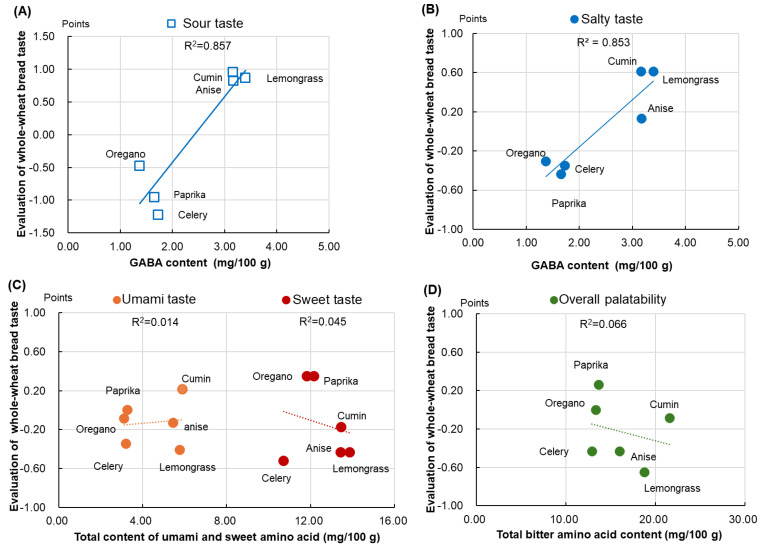
Effects of the free amino acid content on the taste quality of spice-added whole-wheat bread. The effects of (**A**,**B**) GABA, (**C**) umami and sweet amino acid, and (**D**) bitter amino acid contents. Free amino acid content: *n* = 3. Sensory taste of spice-added whole-wheat bread compared with that of the control bread: *n* = 23. Scoring method: Each of the four basic tastes was evaluated using a seven-point scoring system as follows: very strong (+3), strong (+2), slightly strong (+1), no change (0), slightly weak (−1), weak (−2), and very weak (−3). For the overall evaluation, the following seven-point scoring system was used: very tasty (+3), tasty (+2), somewhat tasty (+1), no change (0), somewhat bad (−1), bad (−2), and very bad (−3). Scores: Average values. GABA, γ-aminobutyric acid.

Figure 2 presents the relationship between the free amino acid content and taste quality of the spice-added whole-wheat bread. Taste quality was evaluated through sensory testing using scoring methods and comparing the results with those of the control bread, which received 0 points.

As GABA has the effect of imparting acidity and enhancing saltiness, a regression analysis was conducted on the amount of GABA, acidity, and saltiness (Figure 2A,B). The results showed a positive association between the amount of GABA and the acidity and saltiness of bread (R-squared values: 0.857 and 0.853, respectively). Spices contributing to acidity and saltiness were lemongrass, cumin, and anise, with GABA levels exceeding 3 mg (Figure 2A,B). As presented in Table 1, the threshold for GABA is 2.5 mg/100 g. Therefore, the salty and sour taste of low-salt whole-wheat bread can be attributed to GABA, which is increased by the addition of extracts.

The total free umami amino acid contents were higher in anise, lemongrass, and cumin (approximately 6 mg) than in the other spices; however, the umami ratings for anise and lemongrass were negative, with lemongrass being scored the lowest and the R-squared value was 0.014 (Figure 2C, left). These results indicate that the changes in the amount of free umami amino acids may not be directly correlated with the umami taste of the bread.

The total contents of free sweet amino acids were higher in anise, cumin, and lemongrass (approximately 14 mg) than in the other spices (Figure 2C, right). However, the sweetness rating was negative for all spices, and the R-squared value was 0.045 (Figure 2C). These findings suggest no relationship between the amount of free amino acids added by spices and sweetness rating.

As bitterness conflicts with the good taste of bread, we compared the total content of bitter amino acids with the overall evaluation of the good taste of bread (Figure 2D). The results suggest that the total content of bitter amino acids is not associated with the overall palatability of bread (R-squared value: 0.066); however, the taste quality of lemongrass bread is low (Figure 2D).

### 3.4. Preference for Bread with Spice Extract

**Figure 3:**
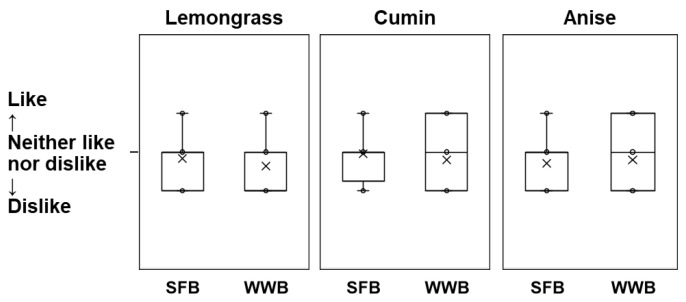
Comparison of the sensory evaluation of spice-added bread and the control bread. The evaluation was performed at three levels—like (+1), neither like nor dislike (0), and dislike (−1). The boxes show the lower quartile and upper quartile values of the data. The lines represent the median values. × represents the average value. The whiskers and circles represent the range of data (minimum value to maximum value), respectively, which were defined by the default settings of the Excel statistical function. SFB: Strong flour bread (*n* = 23); WWB: whole-wheat bread (*n* = 24). Data were analyzed using the Kruskal–Wallis test. Lemongrass: *p* = 0.347; cumin: *p* = 0.411; and anise: *p =* 0.821.

As lemongrass, cumin, and anise influence the sourness, saltiness, and overall palatability of whole-wheat breads, we compared whether the spice-added breads were more liked than the control breads (Figure 3). Lemongrass bread was disliked more than the control bread, regardless of whether it was the strong flour or whole-wheat bread. In contrast, whole-wheat breads with cumin or anise tended to be more preferred than the strong flour breads, although the differences were not significant.

## 4. Discussion

In this study, cumin and anise extracts, which are commonly used in food, were effective in increasing the GABA level in low-salt whole-wheat bread. Both the preference and saltiness of the bread were favorable, indicating that they are useful for reducing the salt content of whole-wheat bread without compromising the taste. In addition, the consumption of 100 g of low-salt whole-wheat bread with these added spices has been suggested to provide approximately 30% of the functional GABA levels recommended for managing hypertension per day [10]. The strong Kitanokaori flour used in this study is characterized by its relatively low free amino acid content and heating flavor components, such as pyrazine, compared to other domestic flours [28]. Nagano et al. [29] blended 90% Kitanokaori wheat flour with 10% Haruyutaka whole-wheat flour, thereby varying the amino acid composition and amount. Thus, when improving the taste and flavor of bread, the increase in the number of amino acids related to taste and flavor should be considered.

One major question addressed in this study pertained to the role of GABA in low-salt whole-wheat breads—specifically, whether ABA is produced in whole-wheat bread by the addition of spice extracts, and if it is produced, how GABA acts as a messenger to other tastes.

GABA is synthesized by GAD, and the free amino acid analysis shows that whole-wheat bread contains six times more GABA than strong flour bread. This finding confirms the presence of endogenous GAD in bread. Regarding endogenous GAD in flour, Lamberts et al. [30] investigated the dynamics of GABA in bread production and found that GABA in bread is synthesized in small amounts from glutamate by GAD in flour. In particular, baker’s yeast is the main contributor to GABA synthesis in bread. Furthermore, the GABA content reportedly increases when GAD is exogenously supplemented [30]. GABA production was also confirmed in wheat germ in a study by Takigawa et al. [13]. In addition, studies focusing on endogenous GAD in baker’s yeast have been reported, with Ando et al. [31,32] examining GABA-enriched baker’s yeast and Diana et al. [33] focusing on GABA levels in bread made with sourdough. The increased GABA production in such bread was suggested to be due to an enzymatic reaction.

In this study, quantification of GABA in the lemongrass, anise, and cumin breads showed that the GABA level increased approximately two-fold, suggesting that the spice extracts affect the GAD level in whole-wheat breads. Although the results are for six spice extracts, some evidence suggests that these spices may activate GAD.

In our previous studies, we purified GAD67 and GAD65, isoforms of GAD expressed in taste bud cells [11,34], from transfected rat brain GAD/DNA in baker’s yeast and *E. coli*, respectively. We performed GAD activity tests using various food extracts, most of which are classified as spices and herbs [8,12]. Our results indicated that the effect of food extracts on GAD activity varied widely; some activated it, whereas others inhibited it [8,12]. Together with the findings in the present in vitro experiments, these findings indicate that spice extracts, such as lemongrass, anise, and cumin, act on the endogenous GAD enzyme present in the germ in whole-wheat flour, consequently increasing GABA levels. This increase, in turn, contributes to the enhanced saltiness of low-salt whole-wheat bread.

We further demonstrated that whole-wheat bread with cumin and anise was preferred over strong flour bread. The effect of free amino acids on sweetness or umami taste in low-salt whole-wheat bread with added spice extracts may arise from a combination of amino acids rather than a single amino acid. A previous study has demonstrated that the presence of a small amount of GABA has a taste-contrast effect on umami (monosodium glutamate [MSG]) [12]. Hence, we plotted the data of the six spice extracts tested and analyzed the correlation between the ratio of glutamate relative to GABA and umami taste (Figure S2). The findings demonstrate the existence of an optimal ratio of glutamate to GABA that maximizes umami taste in spice-added whole-wheat bread. Based on these results, we speculate that GABA in whole-wheat bread may contribute to the umami taste via the taste-contrast effect, akin to the action of salt; nevertheless, further research is warranted to explore the specific type of spices.

In the present study, proline contents increased in the whole-wheat breads to which the extracts of lemongrass and cumin were added. Morita et al. [35] reported that the addition of proline to strong flour for making bread enhanced the umami taste rather than the sweetness. If the amino acid content of the control bread, such as Kitanokaori, could be increased with spice extracts, it might be feasible to improve the taste and flavor of the bread. Nevertheless, further research is required to clarify the role of proline on the taste of whole-wheat breads and the detailed mechanisms underlying the proline dynamics following the addition of lemongrass and cumin extracts during bread production.

Feasible methods for reducing salt levels in bread include decreasing the amount of salt used, substituting potassium chloride (KCl), and employing a combination of KCl and magnesium chloride. These methods can reduce salt levels by 30–50% [36,37]. Maheshwari et al. [38] prepared bread with a reduced sodium level (traditional Indian fried bread) by adding spices, such as cumin and MSG, to ensure a good taste enriched with the flavors of cumin and MSG [38]. In this study, we prepared whole-wheat bread with a 50% reduction in salt content by adding spices commonly used in food rather than chemicals, such as KCl and monosodium glutamate. Notably, the use of spices did not significantly alter the flavor of the bread.

The study has some limitations. The participants of this study were mainly young students; however, if the age range is broadened, there may be differences in taste preferences depending on dietary experiences with whole grains and spices. Furthermore, the detailed mechanism underlying the dynamics of free amino acids following the addition of spices in bread production is still unclear. Extracts of cumin and anise likely stimulate GAD to increase GABA levels; therefore, using these extracts as saltiness enhancers in bread could potentially facilitate the development of whole-wheat bread with reduced salt content.

## 5. Conclusions

This study demonstrated that the addition of small amounts of cumin and anise extracts, which are inexpensive, readily available, and commonly used in food, to whole-wheat bread dough could activate the intrinsic GAD in the germ and increase the level of GABA. This elevation exhibits a positive effect on saltiness perception and overall preference and can effectively reduce salt intake levels. Furthermore, GABA may serve as a functional food item for hypertension prevention. This study provides a theoretical basis for guiding industrial production toward the development of healthy and taste-enriched GABA-mediated low-salt breads using spice extracts.

## Figures and Tables

**Table 1:** Free amino acid categorization according to taste quality.

Taste Quality	Amino Acid	Threshold Value mg/100 g		
		Yoshida et al. ^1^	Tanaka et al. ^2^	Others ^3^
Sour amino acid, enhancing the salty taste	GABA	-	-	2.5
Umami amino acids	Glu	5 ^A^	30 ^a^	12 ^a^
	Asp	3 ^A^	100 ^b^	
Sweet amino acids	Pro	-	300	
	Gln	-	-	
	Ala	60	60	
	Thr	260	260	
	Gly	130	110	
	Ser	150	150	
	Hypro	50	-	
Bitter amino acids	Cys	-	50	
	Arg	50	10	
	Lys	50 ^c^	50 ^c^	
	His	20	20	
	Val	40	150	
	Phe	-	150	
	Ile	90	90	
	Met	30	30	
	Trp	-	-	
	Leu	190	-	
Others	Asn	-	-	
	Tyr	-	-	
	Cys2	-	-	

Abbreviations for the amino acids analyzed in the order of elution: 1. L-Histidine (His), 2. L-Arginine (Arg), 3. L-Hydroxyproline (Hypro), 4. L-Asparagine (Asn), 5. L-Glutamine (Gln), 6. L-Serine (Ser), 7. L-Aspartate (Asp), 8. L-Glutamate (Glu), 9. L-Threonine (Thr), 10. L-Proline (Pro), 11. Glycine (Gly), 12. Gamma-aminobutyric acid (GABA), 13. L-Alanine (Ala), 14. L-Valine (Val), 15. L-Methionine (Met), 16. L-Leucine (Leu), 17. L-Isoleucine (Ile), 18. L-Cystine (Cys2), 19. L-Tryptophan (Trp), 20. L-Phenylalanine (Phe), 21. L-Lysine (Lys), 22. L-Cysteine (Cys), and 23. L-Tyrosine (Try). ^1^ Yoshida et al. [24]; ^2^ Tanaka et al. [21]; ^3^ Others: Hisaki et al. [12], Yamaguchi [22] ^a^ L-Glu･Na; ^b^ L-Asp･Na; ^c^ L-Lys･HCl; ^A^ Sour taste.

**Table 2:** Free amino acid contents of strong flour and whole-wheat breads.

Taste Quality	Amino Acid	SFB mg/100 g	WWB mg/100 g	The Ratio of WWB/SFB
		Means ± SE	Means ± SE	
Sour amino acid, enhancing the salty taste	GABA	0.25 ± 0.02	1.47 ± 0.01	6.0
Umami amino acids	Glu	3.14 ± 0.08	2.21 ± 0.02	0.7
	Asp	0.57 ± 0.01	0.64 ± 0.01	1.1
	Total	3.72 ± 0.08	2.85 ± 0.02	0.8
Sweet amino acids	Pro	1.45 ± 0.07	2.27 ± 0.03	1.6
	Gln	1.09 ± 0.07	2.09 ± 0.04	1.9
	Ala	0.76 ± 0.03	1.10 ± 0.01	1.4
	Thr	0.53 ± 0.01	0.86 ± 0.03	1.6
	Gly	0.46 ± 0.04	1.19 ± 0.01	2.6
	Ser	0.24 ± 0.03	0.52 ± 0.01	2.2
	Hypro	Nd	Nd	-
	Total	4.54 ± 0.11	8.03 ± 0.04	1.8
Bitter amino acids	Cys	2.42 ± 0.10	4.19 ± 0.29	1.7
	Arg	1.24 ± 0.04	3.20 ± 0.05	2.6
	Lys	0.53 ± 0.01	0.67 ± 0.02	1.2
	His	0.49 ± 0.02	1.07 ± 0.04	2.2
	Val	0.37 ± 0.03	0.55 ± 0.03	1.5
	Phe	0.31 ± 0.03	0.47 ± 0.03	1.5
	Ile	0.20 ± 0.01	0.24 ± 0.02	1.2
	Met	0.12 ± 0.01	0.11 ± 0.01	0.9
	Trp	0.08 ± 0.01	2.05 ± 0.03	25.9
	Leu	Nd	Nd	-
	Total	5.77 ± 0.10	12.55 ± 0.26	2.2
Others	Asn	4.01 ± 0.05	5.16 ± 0.03	1.3
	Tyr	0.22 ± 0.02	1.03 ± 0.02	4.8
	Cys2	0.16 ±0.02	0.18 ± 0.00	1.1
Total		18.66 ± 0.32	31.27 ± 0.25	1.6

SFB: Strong flour bread (*n* = 6), WWB: Whole-wheat bread (*n* = 3), Nd: not detected.

## Data Availability

The original contributions presented in the study are included in the article and supplementary materials, further inquiries can be directed to the corresponding author.

## Supplementary Materials

The following supporting information can be downloaded at: https://www.mdpi.com/article/10.3390/foods13121900/s1; Figure S1. Typical flours available in Japan and their uses; Figure S2. Effect of the ratio of glutamate/GABA of spice-added whole-wheat bread on umami taste.
