# Supplementary material for: Contribution of Gamma-Aminobutyric Amino Acid and Free Amino Acids to Low-Salt Whole-Wheat Bread through the Addition of Spice Extracts—An Approach Based on Taste Quality
Source: Foods. 2024 Jun 17;13(12):1900. doi: 10.3390/foods13121900 (PMC11203152; doi:10.3390/foods13121900)
Supplement: Supplementary file 1 [file foods-13-01900-s001.zip › Figure S1.pdf]

Supplementary Materials

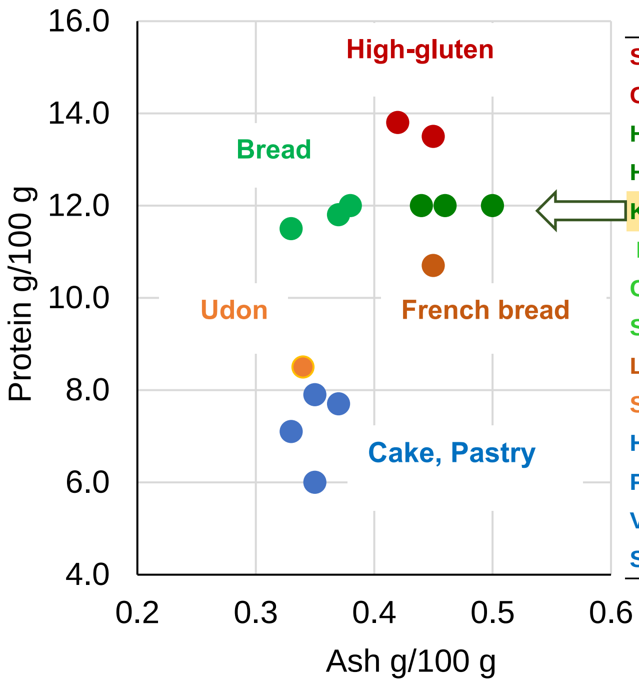

Brand name	Protein g/100 g
Super king	13.8
Golden Yacht	13.5
Haruyutaka	12.0
Haruyokoi	12.0
Kitanokaori	12.0
Eagle	12.0
Camelliya	11.8
Super Camellia	11.5
Lisdool	10.7
Shirotsubaki	8.5
Heart	7.9
Flower	7.7
Violet	7.1
Super Violet	6.0

Japanese Bread Flour (Haruyutaka, Haruyokoi, Kitanokaori)

※Protein and ash content are presented as weight (g) per 100 g of flour.

Figure S1. Typical flours available in Japan and their uses. "Kitanokaori," a hard red winter wheat variety developed by the National Agricultural Research Center for the Hokkaido Region in Japan, was released in January 2003 [15].
