# Supplementary material for: Contribution of Gamma-Aminobutyric Amino Acid and Free Amino Acids to Low-Salt Whole-Wheat Bread through the Addition of Spice Extracts—An Approach Based on Taste Quality
Source: Foods. 2024 Jun 17;13(12):1900. doi: 10.3390/foods13121900 (PMC11203152; doi:10.3390/foods13121900)
Supplement: Supplementary file 1 [file foods-13-01900-s001.zip › Figure S2.pdf]

Supplementary Materials

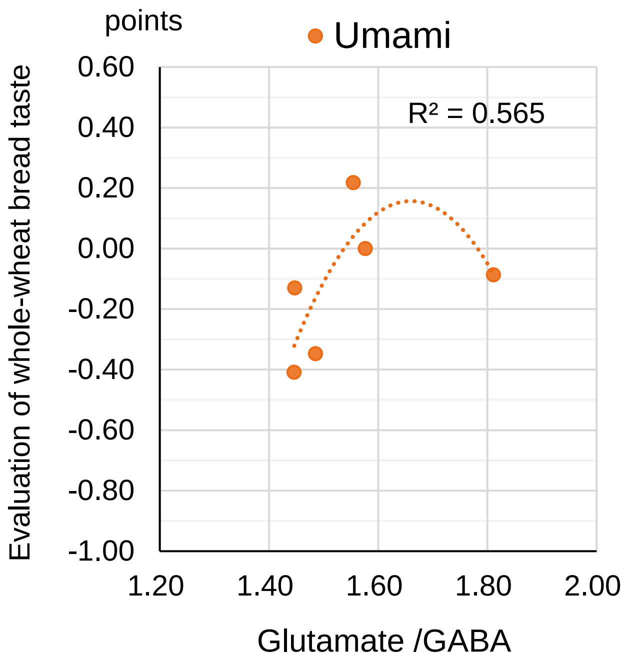

Spice extract	Umami point	Glutamate (mg/100 g)	GABA (mg/100 g)	Glutamate / GABA
Oregano	-0.09	2.48	1.37	1.81
Paprika	0.00	2.61	1.65	1.58
Cumin	0.22	4.91	3.16	1.55
Celery	-0.35	2.57	1.73	1.49
Anise	-0.13	4.59	3.17	1.45
Lemongrass	-0.41	4.91	3.39	1.45

Figure S2. Effect of the ratio of glutamate/GABA of spice-added whole-wheat bread on umami taste. Free amino acid content: n = 3. Sensory taste of spice-added whole-wheat bread compared with that of the control bread: n = 23. Scoring method: The umami taste was evaluated using a seven-point scale from +3 to –3: very strong (+3), strong (+2), slightly strong (+1), no change (0), slightly weak (–1), weak (–2), and very weak (–3). Scores: Average values. The taste-contrast effect: If one taste is strong and the other is very slight, the dominant taste is perceived as stronger. It is the same effect sensed when a pinch of salt is added to kombu dashi.
